# Red Blood Cell Transfusion and Mortality in Trauma Patients: Risk-Stratified Analysis of an Observational Study

**DOI:** 10.1371/journal.pmed.1001664

**Published:** 2014-06-17

**Authors:** Pablo Perel, Tim Clayton, Doug G. Altman, Peter Croft, Ian Douglas, Harry Hemingway, Aroon Hingorani, Katherine I. Morley, Richard Riley, Adam Timmis, Danielle Van der Windt, Ian Roberts, for the PROGRESS Partnership

**Affiliations:** 1 Epidemiology & Population Health Faculty, London School of Hygiene & Tropical Medicine, London, United Kingdom; 2 Centre for Statistics in Medicine, University of Oxford, Oxford, United Kingdom; 3 Arthritis Research UK Primary Care Centre, Keele University, Staffordshire, United Kingdom; 4 Department of Epidemiology and Public Health, University College London, London, United Kingdom; 5 School of Population and Global Health, The University of Melbourne, Melbourne, Australia; 6 School of Health and Population Sciences, University of Birmingham, Birmingham, United Kingdom; 7 London Chest Hospital, London, United Kingdom; University of Cambridge, United Kingdom

## Abstract

**Background:**

Haemorrhage is a common cause of death in trauma patients. Although transfusions are extensively used in the care of bleeding trauma patients, there is uncertainty about the balance of risks and benefits and how this balance depends on the baseline risk of death. Our objective was to evaluate the association of red blood cell (RBC) transfusion with mortality according to the predicted risk of death.

**Methods and Findings:**

A secondary analysis of the CRASH-2 trial (which originally evaluated the effect of tranexamic acid on mortality in trauma patients) was conducted. The trial included 20,127 trauma patients with significant bleeding from 274 hospitals in 40 countries. We evaluated the association of RBC transfusion with mortality in four strata of predicted risk of death: <6%, 6%–20%, 21%–50%, and >50%. For this analysis the exposure considered was RBC transfusion, and the main outcome was death from all causes at 28 days. A total of 10,227 patients (50.8%) received at least one transfusion. We found strong evidence that the association of transfusion with all-cause mortality varied according to the predicted risk of death (*p-*value for interaction <0.0001). Transfusion was associated with an increase in all-cause mortality among patients with <6% and 6%–20% predicted risk of death (odds ratio [OR] 5.40, 95% CI 4.08–7.13, *p<*0.0001, and OR 2.31, 95% CI 1.96–2.73, *p<*0.0001, respectively), but with a decrease in all-cause mortality in patients with >50% predicted risk of death (OR 0.59, 95% CI 0.47–0.74, *p<*0.0001). Transfusion was associated with an increase in fatal and non-fatal vascular events (OR 2.58, 95% CI 2.05–3.24, *p<*0.0001). The risk associated with RBC transfusion was significantly increased for all the predicted risk of death categories, but the relative increase was higher for those with the lowest (<6%) predicted risk of death (*p-*value for interaction *<*0.0001). As this was an observational study, the results could have been affected by different types of confounding. In addition, we could not consider haemoglobin in our analysis. In sensitivity analyses, excluding patients who died early; conducting propensity score analysis adjusting by use of platelets, fresh frozen plasma, and cryoprecipitate; and adjusting for country produced results that were similar.

**Conclusions:**

The association of transfusion with all-cause mortality appears to vary according to the predicted risk of death. Transfusion may reduce mortality in patients at high risk of death but increase mortality in those at low risk. The effect of transfusion in low-risk patients should be further tested in a randomised trial.

**Trial registration:**

www.ClinicalTrials.gov NCT01746953

*Please see later in the article for the Editors' Summary*

## Abstract 2

Using a large multicentre cohort, Pablo Perel and colleagues evaluate the association of red blood cell transfusion with mortality according to the predicted risk of death for trauma patients.

*Please see later in the article for the Editors' Summary*

## Editors' Summary

**Background:**

Trauma—a serious injury to the body caused by violence or an accident—is a major global health problem. Every year, injuries caused by traffic collisions, falls, blows, and other traumatic events kill more than 5 million people (9% of annual global deaths). Indeed, for people between the ages of 5 and 44 years, injuries are among the top three causes of death in many countries. Trauma sometimes kills people through physical damage to the brain and other internal organs, but hemorrhage (serious uncontrolled bleeding) is responsible for 30%–40% of trauma-related deaths. Consequently, early trauma care focuses on minimizing hemorrhage (for example, by using compression to stop bleeding) and on restoring blood circulation after blood loss (health-care professionals refer to this as resuscitation). Red blood cell (RBC) transfusion is often used for the management of patients with trauma who are bleeding; other resuscitation products include isotonic saline and solutions of human blood proteins.

**Why Was This Study Done?:**

Although RBC transfusion can save the lives of patients with trauma who are bleeding, there is considerable uncertainty regarding the balance of risks and benefits associated with this procedure. RBC transfusion, which is an expensive intervention, is associated with several potential adverse effects, including allergic reactions and infections. Moreover, blood supplies are limited, and the risks from transfusion are high in low- and middle-income countries, where most trauma-related deaths occur. In this study, which is a secondary analysis of data from a trial (CRASH-2) that evaluated the effect of tranexamic acid (which stops excessive bleeding) in patients with trauma, the researchers test the hypothesis that RBC transfusion may have a beneficial effect among patients at high risk of death following trauma but a harmful effect among those at low risk of death.

**What Did the Researchers Do and Find?:**

The CRASH-2 trail included 20,127 patients with trauma and major bleeding treated in 274 hospitals in 40 countries. In their risk-stratified analysis, the researchers investigated the effect of RBC transfusion on CRASH-2 participants with a predicted risk of death (estimated using a validated model that included clinical variables such as heart rate and blood pressure) on admission to hospital of less than 6%, 6%–20%, 21%–50%, or more than 50%. That is, the researchers compared death rates among patients in each stratum of predicted risk of death who received a RBC transfusion with death rates among patients who did not receive a transfusion. Half the patients received at least one transfusion. Transfusion was associated with an increase in all-cause mortality at 28 days after trauma among patients with a predicted risk of death of less than 6% or of 6%–20%, but with a decrease in all-cause mortality among patients with a predicted risk of death of more than 50%. In absolute figures, compared to no transfusion, RBC transfusion was associated with 5.1 more deaths per 100 patients in the patient group with the lowest predicted risk of death but with 11.9 fewer deaths per 100 patients in the group with the highest predicted risk of death.

**What Do These Findings Mean?:**

These findings show that RBC transfusion is associated with an increase in all-cause deaths among patients with trauma and major bleeding with a low predicted risk of death, but with a reduction in all-cause deaths among patients with a high predicted risk of death. In other words, these findings suggest that the effect of RBC transfusion on all-cause mortality may vary according to whether a patient with trauma has a high or low predicted risk of death. However, because the participants in the CRASH-2 trial were not randomly assigned to receive a RBC transfusion, it is not possible to conclude that receiving a RBC transfusion actually increased the death rate among patients with a low predicted risk of death. It might be that the patients with this level of predicted risk of death who received a transfusion shared other unknown characteristics (confounders) that were actually responsible for their increased death rate. Thus, to provide better guidance for clinicians caring for patients with trauma and hemorrhage, the hypothesis that RBC transfusion could be harmful among patients with trauma with a low predicted risk of death should be prospectively evaluated in a randomised controlled trial.

**Additional Information:**

Please access these websites via the online version of this summary at http://dx.doi.org/10.1371/journal.pmed.1001664.

- This study is further discussed in a *PLOS Medicine* Perspective by Druin Burch
- The World Health Organization provides information on injuries and on violence and injury prevention (in several languages)
- The US Centers for Disease Control and Prevention has information on injury and violence prevention and control
- The National Trauma Institute, a US-based non-profit organization, provides information about hemorrhage after trauma and personal stories about surviving trauma
- The UK National Health Service Choices website provides information about blood transfusion, including a personal story about transfusion after a serious road accident
- The US National Heart, Lung, and Blood Institute also provides detailed information about blood transfusions
- MedlinePlus provides links to further resources on injuries, bleeding, and blood transfusion (in English and Spanish)
- More information in available about CRASH-2 (in several languages)

## Introduction

Haemorrhage is a leading cause of death in trauma patients, responsible for approximately 30% to 40% of trauma-related deaths [1],[2]. Although red blood cell (RBC) transfusion is often used in the management of bleeding trauma patients, there is considerable uncertainty regarding the balance of risks and benefits [3],[4].

RBC transfusion is a scarce and expensive intervention with potential adverse effects, including allergic reaction, transfusion-related lung injury, graft versus host disease, and infection. Furthermore, supplies of blood are lower, and the risks from transfusion higher, in low- and middle-income countries, where most bleeding deaths occur [5].

A systematic review showed that RBC transfusion is associated with increased morbidity and mortality in critically ill patients, including trauma patients [6]. Nevertheless, the included studies were observational, and it is likely that some of the effect observed was due to confounding by indication, with transfusion being offered to more severely ill patients. A more recent systematic review of randomised trials evaluated the effect of different haemoglobin or haematocrit thresholds for blood transfusion in haemodynamically stable critically ill patients. It found that a more restrictive approach (transfusion only when haemoglobin levels were below 70 or 80 g/l) reduced in-hospital mortality without any increase in adverse events [7].

However, most RBC transfusion in trauma patients occurs early after hospital admission, when haematocrit level is not a reliable indicator of the extent of bleeding, and clinicians must use physical signs, diagnostic tests, and clinical judgment to decide whether or not a RBC transfusion is indicated [8].

It is possible that the effect of RBC transfusion on mortality depends on the underlying risk. We hypothesized that there may be a beneficial effect among patients at high risk of death but a harmful effect in those patients at low risk of death. Even if the relative effect is similar, the absolute effect and cost-effectiveness could vary according to underlying risk, and so a stratified approach to RBC transfusion might be justified. To the best of our knowledge, this hypothesis has not been tested before in trauma patients. Using a large international cohort of trauma patients with bleeding, we evaluated the association of RBC transfusion with mortality according to the predicted risk of death.

## Methods

### Ethics Statement

This study received ethics approval from the London School of Hygiene & Tropical Medicine.

### Aims

The primary objective of the study was to evaluate the association of RBC transfusion with all-cause mortality at 28 days (or hospital discharge) according to predicted risk of death at hospital admission. The secondary objective was to evaluate the association of RBC transfusion with fatal and non-fatal vascular occlusive events.

### Sample

The study cohort included all patients from the CRASH-2 clinical trial. The trial included 20,127 trauma patients with, or at risk of, significant bleeding within 8 h of injury, and evaluated the effect of tranexamic acid on all-cause mortality. The trial was undertaken in 274 hospitals in 40 countries. Detailed information on the methods and results of the CRASH-2 trial have been published previously [9].

### Outcomes

The primary outcome of this analysis was death from all causes stratified by baseline risk. We also reported specific causes of death (bleeding, head injury, multi-organ failure, myocardial infarction, stroke, pulmonary embolism, and other causes), and we conducted a secondary analysis exploring the association of RBC transfusion with fatal and non-fatal vascular occlusive events including myocardial infarction, stroke, deep vein thrombosis, and pulmonary embolism. All events were measured at 28 days or hospital discharge. Cause of death was defined by the investigators using their clinical judgment.

### Interventions and Comparisons

We compared the association of RBC transfusion with the outcomes versus that of no RBC transfusion. For this analysis we compared two groups: those who received at least one RBC transfusion (transfused) versus those patients who did not receive any RBC transfusion (non-transfused).

### Statistical Analysis

The characteristics of patients were tabulated and compared according to whether the patient underwent a transfusion. Univariable comparisons were made using a logistic regression model by treating each variable as a categorical or continuous co-variate as appropriate.

For each patient we estimated the predicted risk of death from all causes using a validated model, and categorised patients into four pre-specified strata (<6%, 6%–20%, 21%–50%, and >50%). The prognostic model we used was developed using 20,127 trauma patients with, or at risk of, significant bleeding within 8 h of injury. The model development was conducted with a backward stepwise approach, and the predictors included in the final model were Glasgow Coma Scale, age, heart rate, systolic blood pressure, time since injury, type of injury, and geographical region. Full details of model development and validation have been published elsewhere [10] (see Text S1). Although risk is a continuous variable, we decided to use risk categories for simplifying its use in clinical practice. The risk categories used were identical to the ones reported in the original prognostic model, and the cutoffs were decided with the feedback from prognostic model users and by looking at previous publications [10].

The number of patients and number of deaths were tabulated by transfusion status. Odds ratios (ORs) and risk differences, together with 95% confidence intervals, comparing RBC transfusion to no RBC transfusion were calculated within each of the pre-specified risk categories as defined previously [10]. Interaction tests were conducted using logistic regression to formally assess whether the impact of RBC transfusion differed according to underlying risk, with risk considered as a continuous variable.

Because RBC transfusion practices vary and could be associated with different risks according to the region of the world, we also examined the association with death from all causes separately for four geographical regions.

To identify a potential non-linear interaction between transfusion and baseline risk, patients were also categorised into ten risk groups containing approximately one-tenth of the primary outcome each, and the association of RBC transfusion with death from all causes was evaluated within each of these categories.

We conducted complete case analyses, as the amount of missing data was very low (1%).

#### Sensitivity analyses

To take into account a potential survival bias we also reported the association of RBC transfusion with all-cause mortality excluding patients who died on day “0” (first day of hospital arrival).

To examine the impact of possible confounding by indication, we calculated propensity scores for all patients using logistic regression, with blood transfusion as the outcome. Factors included in the model were those likely to influence the decision to transfuse, including age, gender, income region (high, middle, or low), systolic blood pressure, heart rate, respiratory rate, Glasgow Coma Scale, type of injury (penetrating or blunt), time since injury, and tranexamic acid use. The distribution of propensity scores amongst all transfused and non-transfused patients was then compared, and we excluded all patients with scores in the upper and lower 5% of the score distribution. Any patients whose propensity scores were outside the overlapping area of the distributions of transfused and non-transfused patients were also excluded, to avoid making comparisons between patients with too many underlying differences. With this reduced study population, we then evaluated the association of transfusion with all-cause mortality according to the predicted risk of death in each of the pre-specified mortality strata, adjusting by the propensity score (as a continuous variable).

Finally, to take into account potential confounding by geographical variation in the types of blood products used for transfusion, we adjusted the comparison within each predicted risk group by use of platelets, fresh frozen plasma, and cryoprecipitate and by country using logistic regression.

Stata Statistical Software Release 11 (StataCorp) was used for the analysis.

## Results

The baseline characteristics of CRASH-2 trial patients according to their RBC transfusion status are displayed in Table 1. A total of 10,227 patients (50.8%) received RBC transfusion. Patients from high-income countries, and those who arrived at hospital more than 3 h after the injury, had lower systolic blood pressure or Glascow Coma Score, had higher heart rate or respiratory rate, or had blunt injury were more likely to receive RBC transfusion (*p<*0.0001 for all comparisons, except *p = *0.010 for blunt versus penetrating injuries). Patients in the lowest predicted risk of death category (<6%) were less likely to receive RBC transfusions.

**Table 1:** Baseline characteristics by transfusion status.

Characteristic	Subcategory	Number with Missing Values	All Patients	Transfusion		
				Yes	No	*p*-Value
**Total**		270	20,127	10,227 (50.8%)	9,900	—
**Country income**	High	0	414	343 (82.9%)	71	—
	Middle	0	19,408	9,715 (50.1%)	9,693	<0.0001
	Low	0	305	169 (55.4%)	136	<0.0001
**Tranexamic acid**	Placebo	0	10,067	5,160 (51.3%)	4,907	—
	Active	0	10,060	5,067 (50.4%)	4,993	0.21
**Time from injury to arrival at hospital**	≤3 h	8	13,485	6,506 (48.2%)	6,979	—
	>3 h		6,634	3,715 (56.0%)	2,919	<0.0001
**Age (years)**		1	30 (24 to 43)	31 (21 to 43)	30 (24 to 43)	0.9
**Systolic blood pressure (mm Hg)**		28	91 (80 to 110)	90 (80 to 100)	100 (90 to 120)	<0.0001
**Respiratory rate (per min)**		186	22 (20 to 26)	22 (20 to 28)	22 (19 to 26)	<0.0001
**Heart rate (per min)**		137	105 (90 to 120)	110 (96 to 120)	100 (88 to 112)	<0.0001
**Glasgow Coma Scale**		23	15 (11 to 15)	14 (10 to 15)	15 (12 to 15)	<0.0001
**Penetrating injury**	No	0	13,605	6,998 (51.4%)	6,607	—
	Yes	0	6,522	3,229 (49.5%)	3,293	0.01
**Mortality at 28 days**	No	0	17,051	8,206 (48.1%)	8,845	<0.0001
	Yes	0	3,076	2,021 (65.7%)	1,055	
**Predicted risk of death** a	<6%		8,706	3,406 (39.1%)	5,300	—
	6% to 20%		6,850	3,905 (57.0%)	2,945	<0.0001
	>20% to 50%		2,758	1,761 (63.9%)	997	<0.0001
	>50%		1,543	960 (62.2%)	583	<0.0001

Data are presented as number, number (percent), or median (interquartile range).

a From a logistic regression model fitting each covariate as a categorical or continuous variable. Predicted risk of death was not calculated for those with missing values.

All-cause mortality was higher in patients who received RBC transfusion (Table 2). A total of 2,021 (19.8%) patients who received a RBC transfusion died, while 1,055 (10.7%) patients who did not receive RBC transfusion died (OR 2.06, 95% CI 1.91–2.24, *p<*0.0001). Deaths from bleeding (OR 3.16, 95% CI 2.74–3.64, *p<*0.0001), multi-organ failure (OR 3.44, 95% CI 2.74–4.30, *p<*0.0001), myocardial infarction (OR 3.05, 95% CI 1.30–7.13, *p = *0.010), and other causes (OR 2.80, 95% CI 2.12–3.69, *p<*0.0001) were more frequent in patients who received a RBC transfusion than in those who did not receive one.

**Table 2:** Clinical outcomes by red blood cell transfusion.

Outcome	Transfusion (*n = *10,227)	No Transfusion (*n = *9,900)	Total (*n = *20,127)	*p*-Value
**All-cause mortality**	2,021 (19.8%)	1,055 (10.7%)	3,076 (15.3%)	<0.0001
**Cause-specific mortality**				
Bleeding	803 (7.9%)	260 (2.6%)	1,063 (5.3%)	<0.0001
Head injury	624 (6.1%)	600 (6.1%)	1,224 (6.1%)	0.9
Multi-organ failure	343 (3.4%)	99 (1.0%)	442 (2.2%)	<0.0001
Myocardial infarction	22 (0.2%)	7 (0.1%)	29 (0.1%)	0.01
Stroke	7 (0.1%)	6 (0.1%)	13 (0.1%)	0.83
Pulmonary embolism	25 (0.2%)	14 (0.1%)	39 (0.2%)	0.1
Other causes	197 (1.9%)	69 (0.7%)	266 (1.3%)	<0.0001
**Other outcomes (fatal and non-fatal)**				
Myocardial infarction	67 (0.7%)	23 (0.2%)	90 (0.4%)	<0.0001
Stroke	79 (0.8%)	44 (0.4%)	123 (0.6%)	0.003
Pulmonary embolism	109 (1.1%)	34 (0.3%)	143 (0.7%)	<0.0001
Non-fatal deep vein thrombosis	69 (0.7%)	12 (0.1%)	81 (0.4%)	<0.0001
Vascular occlusive events^a^	267 (2.6%)	102 (1.0%)	369 (1.8%)	<0.0001

Data are presented as number (percent) of patients.

a Myocardial infarction, stroke, pulmonary embolism, or deep vein thrombosis.

A total of 267 (2.6%) patients who received RBC transfusion had a fatal or non-fatal vascular occlusive event, in comparison to 102 (1.0%) of those patients who did not receive a RBC transfusion (OR 2.58, 95% CI 2.05–3.24, *p<*0.0001).

As shown in Table 3 we found strong evidence that the association of RBC transfusion with all-cause mortality differed according to the predicted risk of death (*p-*value for interaction *<*0.0001). A total of 270 patients were excluded from this analysis because at least one variable of the prognostic model was missing (Table S1 provides details of patient characteristics for individuals with missing data). The risk of all-cause mortality associated with RBC transfusion was increased in patients with <6% predicted risk of death, (217 [6.4%] in transfused group versus 66 [1.2%] in non-transfused group; OR 5.40, 95% CI 4.08–7.13, *p<*0.0001). RBC transfusion was also associated with an increase in all-cause mortality in patients with 6%–20% predicted risk of death (591 [15.1%] in transfused group versus 211 [7.2%] in non-transfused group; OR 2.31, 95% CI 1.96–2.73, *p<*0.0001). Among patients with a predicted risk of death of 21%–50%, all-cause mortality was similar in the two groups (557 [31.6%] in transfused group versus 334 [33.5%] in non-transfused group; OR 0.92, 95% CI 0.78–1.08, *p = *0.31), while the risk of all-cause mortality was significantly decreased with RBC transfusion in patients with >50% predicted risk of death (566 [59%] in transfused group versus 413 [70.8%] in non-transfused group; OR 0.59, 95% CI 0.47–0.74, *p<*0.0001).

**Table 3:** Mortality by category of predicted risk of death and red blood cell transfusion.

Predicted Risk of Death^a^	Deaths according to Transfusion Status of Patient		OR (95% CI)	Risk Difference (95% CI)	*p*-Value
	Transfusion	No Transfusion			
<6%	217/3,406 (6.4%)	66/5,300 (1.2%)	5.40 (4.08 to 7.13)	5.1% (4.3% to 6.0%)	<0.0001
6%–20%	591/3,905 (15.1%)	211/2,945 (7.2%)	2.31 (1.96 to 2.73)	8.0% (6.5% to 9.4%)	<0.0001
21%–50%	557/1761 (31.6%)	334/997 (33.5%)	0.92 (0.78 to 1.08)	−1.9% (−5.5% to 1.8%)	0.31
>50%	566/960 (59.0%)	413/583 (70.8%)	0.59 (0.47 to 0.74)	−11.9% (−16.7% to −7.1%)	<0.0001

Interaction between RBC transfusion and predicted risk of death on the OR, *p<*0.0001 (chi-square * = *227 with one degree of freedom).

a Risk group determined according to model published in [10].

In absolute terms, there were 5.1 (95% CI 4.3 to 6.0) more deaths per 100 patients associated with RBC transfusion in the group with the lowest predicted risk of death but 11.9 (95% CI 7.1 to 16.7) fewer deaths per 100 patients associated with RBC transfusion in the group with the highest predicted risk.

The sensitivity analysis (excluding 1,086 patients who died at day 0) showed similar results, indicating that the association of RBC transfusion with all-cause mortality differed according to the predicted risk of death (*p-*value for interaction *<*0.0001) (Table 4). Propensity score analysis (excluding 2,011 patients with extreme propensity score values) showed similar results, with strong evidence of interaction of the association of RBC transfusion with all-cause mortality according to the predicted risk of death (*p-*value for interaction *<*0.0001) (Table 5). The sensitivity analysis adjusting for use of platelets, fresh plasma, and cryoprecipitate and for country also showed a similar pattern and strong evidence of interaction (Table 6).

**Table 4:** Mortality by category of predicted risk of death and red blood cell transfusion excluding deaths on day 0.

Predicted Risk of Death^a^	Deaths according to Transfusion Status of Patient		OR (95% CI)	Risk Difference (95% CI)	*p*-Value
	Transfusion	No Transfusion			
<6%	169/3,358 (5.0%)	47/5,281 (0.9%)	5.90 (4.26 to 8.18)	4.1% (3.4% to 4.9%)	<0.0001
6%–20%	431/3,745 (11.5%)	122/2,856 (4.3%)	2.91 (2.37 to 3.59)	7.2% (6.0% to 8.5%)	<0.0001
21%–50%	406/1,610 (25.2%)	198/861 (23.0%)	1.13 (0.93 to 1.37)	2.2% (−1.3% to 5.7%)	0.22
>50%	370/764 (48.4%)	200/370 (54.1%)	0.80 (0.62 to 1.02)	−5.6% (−11.8% to 0.6%)	0.076

Interaction between RBC transfusion and predicted risk of death on the OR, *p<*0.0001 (chi-square * = *150 with one degree of freedom).

a Risk group determined according to model published in [10].

**Table 5:** Mortality by category of predicted risk of death and red blood cell transfusion adjusted for propensity score.

Predicted Risk of Death^a^	Deaths according to Transfusion Status of Patient		OR (95% CI)	Risk Difference (95% CI)	*p*-Value
	Transfusion	No Transfusion			
<6%	203/3,128 (6.5%)	61/4,633 (1.3%)	4.87 (3.62 to 6.55)	5.0% (4.0% to 5.9%)	<0.0001
6%–20%	558/3,758 (14.8%)	205/2,874 (7.1%)	2.22 (1.86 to 2.63)	7.5% (6.0% to 9.0%)	<0.0001
21%–50%	462/1,450 (31.9%)	288/909 (31.7%)	1.08 (0.90 to 1.30)	1.8% (−2.2% to 5.7%)	0.42
>50%	394/644 (61.2%)	314/449 (69.9%)	0.69 (0.53 to 0.90)	−8.3% (−14.1% to −2.5%)	0.006

Interaction between RBC transfusion and predicted risk of death on the OR, *p<*0.0001 (chi-square * = *151).

a Risk group determined according to model published in [10].

**Table 6:** Mortality by category of predicted risk of death and red blood cell transfusion (adjusted analysis).

Predicted Risk of Death^a^	Deaths according to Transfusion Status of Patient		OR^b^ (95% CI)	*p*-Value
	Transfusion	No Transfusion		
<6%	217/3,346 (6.5%)	66/5,191 (1.3%)	3.68 (2.71 to 5.01)	<0.0001
6%–20%	591/3,750 (15.8%)	211/2,853 (7.4%)	1.92 (1.59 to 2.30)	<0.0001
21%–50%	555/1,761 (31.7%)	332/993 (33.4%)	0.95 (0.78 to 1.16)	0.62
>50%	559/947 (59.0%)	403/573 (70.3%)	0.82 (0.62 to 1.07)	<0.0001

Interaction between RBC transfusion and predicted risk of death on the OR, *p<*0.0001 (chi-square * = *188 with one degree of freedom).

a Risk group determined according to model published in [10].

b OR adjusted for country as well as use of platelets (*n = *806), fresh frozen plasma (*n = *2,633), and cryoprecipitate (*n = *392). In total, 2,726 (13.5%) patients received one of these blood products.

**Figure 1:**
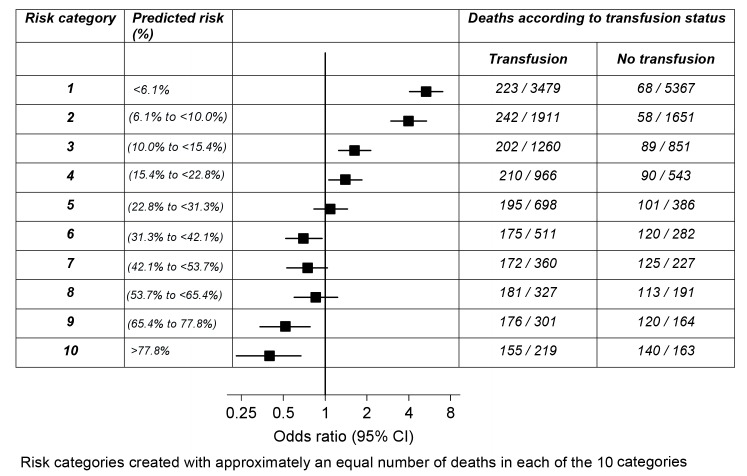
Odds ratio of death for transfusion compared to no transfusion by risk category.

To explore the association of RBC transfusion with all-cause mortality further, we created ten groups of predicted risk of death containing approximately one-tenth of the primary outcome each. As can be seen in Figure 1, RBC transfusion showed a trend from a positive association (harmful) to a negative association (beneficial) with all-cause mortality according to predicted risk of death. RBC transfusion was associated with an increase in all-cause mortality at low predicted risk of death and a decrease in all-cause mortality at high predicted risk of death. The change in direction of the association of transfusion (from harmful to beneficial) with all-cause mortality occurred around a predicted risk of death of about 25%.

We found strong evidence that the association of RBC transfusion with all-cause mortality differed according to the predicted risk of death (*p-*value for interaction *<*0.0001) for each of geographical regions considered (Table 7). Although effect estimates and confidence intervals varied by geographical region, we found the same pattern of association of RBC transfusion and all-cause mortality (positive at low predicted risk of death and negative at high predicted risk of death).

**Table 7:** Mortality with red blood cell transfusion by risk category and geographical region.

Predicted Risk of Death	OR (95% CI)^a^ by Geographical Region			
	Asia	Central and South America	Africa	Europe, North America, and Australasia
<6%	4.94 (2.86 to 8.54)	6.55 (4.16 to 10.32)	2.92 (1.67 to 5.10)	13.01 (4.42 to 38.37)
6%–20%	2.31 (1.74 to 3.07)	2.01 (1.47 to 2.75)	1.77 (1.30 to 2.40)	5.55 (3.15 to 9.79)
21%–50%	1.06 (0.83 to 1.35)	0.80 (0.55 to 1.15)	0.49 (0.33 to 0.71)	1.48 (0.90 to 2.44)
>50%	0.87 (0.62 to 1.22)	0.67 (0.40 to 1.12)	0.44 (0.26 to 0.73)	0.45 (0.27 to 0.76)
**Deaths/total**	1,158/7,250 (16.0%)	737/5,173 (14.2%)	707/4,761 (14.8%)	353/2,673 (13.2%)

Interaction between RBC transfusion and predicted risk of death on the OR, *p<*0.0001 for each continent grouping.

a OR for RBC transfusion versus no RBC transfusion. Risk group determined according to model published in [10].

We also found strong evidence that the association of RBC transfusion with vascular occlusive events differed according to the predicted risk of death (*p-*value for interaction *<*0.0001) (Table 8). The risk associated with RBC transfusion was significantly increased for all the predicted risk of death categories, but the relative increase was higher for those with the lowest predicted risk of death. The OR of vascular occlusive events associated with RBC transfusion was 4.92 (95% CI 2.80–8.65, *p<*0.0001) in patients with <6% predicted risk of death, 1.66 (95% CI 1.13–2.46, *p = *0.009) in patients with 6%–20% predicted risk of death, 1.80 (95% CI 1.16–2.80, *p = *0.006) in patients with 21%–50% predicted risk of death, and 1.58 (95% CI 0.93–2.68, *p = *0.081) in patients with >50% predicted risk of death

**Table 8:** Vascular occlusive events (fatal and non-fatal) by category of predicted risk of death and red blood cell transfusion.

Predicted Risk of Death^a^	Vascular Occlusive Events according to Transfusion Status of Patient		OR (95% CI)	Risk Difference (95% CI)	*p*-Value
	Transfusion	No Transfusion			
<6%	50/3,406 (1.5%)	16/5,300 (0.3%)	4.92 (2.80 to 8.65)	1.2% (0.7% to 1.6%)	<0.0001
6%–20%	81/3,905 (2.1%)	37/2,945 (1.3%)	1.66 (1.13 to 2.46)	0.8% (0.2% to 1.4%)	0.009
21%–50%	84/1,761 (4.8%)	27/997 (2.7%)	1.80 (1.16 to 2.80)	2.1% (0.6% to 3.5%)	0.006
>50%	51/960 (5.3%)	20/583 (3.4%)	1.58 (0.93 to 2.68)	1.9% (−0.2% to 3.9%)	0.081

Interaction between RBC transfusion and predicted risk of death on the OR, *p = *0.013.

a Risk group determined according to model published in [10].

## Discussion

### Main Findings

The association of blood transfusion with all-cause mortality appears to vary according to the predicted risk of death. We found that in patients with a predicted risk of ≤20%, transfusion was associated with an increase in all-cause mortality, while in those patients with high predicted risk of death (>50%), transfusion was associated with reduced mortality. This pattern from harmful to beneficial association was also found when the association of transfusion with mortality was analysed in ten risk categories, and when we analysed patients from different geographical regions separately. In spite of these findings, because of potential biases inherent in this observational study, our findings should be considered cautiously.

Because an increase in vascular occlusive events was hypothesized as one of the possible mechanisms by which RBC transfusion might be harmful, we conducted a stratified analysis for these outcomes [11],[12]. Although we found that the association of transfusion with fatal and non-fatal vascular occlusive events varies according to the predicted risk of death, transfusion was positively associated with vascular occlusive events (harmful) across all risk strata regardless of the predicted risk of death category.

### Strengths and Limitations

Our study has a number of strengths. The CRASH-2 trial was a prospective cohort of bleeding trauma patients, with standardised collection of data on prognostic factors, a large sample size, few missing data, and low loss to follow-up [9]. It included hospitals from low-, middle-, and high-income countries. The prognostic model used in this analysis has shown good performance when externally validated [10]. The study hypothesis was pre-specified, including the risk strata and the direction of the association of transfusion according to the predicted risk of death. The study protocol was registered.

On the other hand, our study has serious limitations. Although our data were from a randomised clinical trial, blood transfusion was not a randomised intervention, and therefore our inferences are vulnerable to confounding [13]. Potential confounding could be suspected because baseline characteristics for transfused and non-transfused patients were different, and those receiving transfusion were at a higher risk of death due to bleeding. Furthermore, there is the possibility of biases acting in different directions depending on the predicted risk of death. For example, in the high-risk group (>50% risk of death), the negative association (beneficial) of RBC transfusion with all-cause mortality could be due to survival bias, since only those who survive are eligible to receive a transfusion [14]. Unfortunately, it was not possible to conduct a time-updated model whereby the period before transfusion was taken into account, since the time of the transfusion was not recorded. Nonetheless, when we attempted to avoid this bias by limiting the analysis to those patients who survived beyond day 0 and therefore had the same opportunity to be transfused, the interaction remained strong.

Conversely, among the low-risk patients, those receiving RBC transfusion might have been at higher risk of death (“confounding by indication”). Propensity scores are useful in observational studies, as they help the researcher to determine whether groups of users and non-users are comparable, and have the potential to reduce confounding by indication [15]. When we conducted an analysis using propensity scores, the results were similar. One potential limitation of using this analytical approach in our study is that there might be a time gap between the variables used in the propensity score (recorded at hospital admission) and the transfusion indication, and this time gap could result in patients being classified as lower risk than they are at the actual time of transfusion. However, the variables included in the propensity scores have been shown to be good predictors of 28-d mortality (which is the transfusion window included in this analysis), so the potential of “misclassifying” to a lower risk category a large proportion of patients using this approach is low [10].

Another limitation of our study is that we could not consider haemoglobin in our analysis. However, our analysis is still informative for current clinical practice, as the indications for a large proportion of RBC transfusions in trauma patients early after hospital admission are based on clinical signs (such as the ones included in our prognostic model) rather than on haemoglobin levels. Furthermore, as mentioned above, the clinical signs included in our prognostic model, such as heart rate and blood pressure, have been shown to be highly predictive of adverse outcomes in patients with trauma and bleeding, and specifically the prognostic model used in our analysis has shown good predictive performance [10].

Finally, the association of blood transfusion with all-cause mortality could have been influenced by the type of blood product received (i.e., whole blood or RBCs) in different countries. Although we did not have this information available, the same pattern and strong evidence for interaction according to baseline risk was found in all the geographical regions. Furthermore, when we further adjusted by use of platelets, fresh frozen plasma, and cryoprecipitate and by country, results were similar.

### Comparison with Previous Studies

Previous studies have shown that RBC transfusions are associated with an increased risk of complications in trauma patients. A systematic review evaluating the association of RBC transfusion with mortality in critically ill patients identified 45 observational studies, and in 42 of them the risks of RBC transfusion outweighed the benefits [6]. The studies included were observational and therefore prone to different types of bias, and, importantly, they did not analyse the association of RBC transfusion with mortality according to baseline risk.

The findings from another systematic review that evaluated the effect of liberal versus restricted transfusion thresholds (haemoglobin or haematocrit triggers) in critically ill patients support the use of restrictive transfusion triggers (haemoglobin levels between 70 and 80 g/l) [7]. Nonetheless, haematocrit level is not a reliable indicator of the extent of bleeding in the early hours after hospital admission, when a substantial proportion of RBC transfusions occur, and clinicians instead use clinical signs and their clinical judgment to decide whether or not to mandate a RBC transfusion. To the best of our knowledge, this is the first study to evaluate the association of RBC transfusion with all-cause mortality stratified by predicted risk of death, using simple clinical variables routinely available at hospital admission.

### Implications for Practice and Research

Current recommendations for trauma and critically ill patients state that transfusion is indicated for patients in “haemorrhagic shock” or who are haemodynamically unstable, and that a restrictive strategy (transfusion when haemoglobin *<*70 g/l) is as effective as a liberal strategy (transfusion when haemoglobin *<*100 g/l) for haemodynamically stable patients [16],[17]. It is important to highlight that only a small proportion of trauma patients would present with haemorrhagic shock, and the vast majority of trauma patients might be unstable but not at very high risk of death [18],[19]. Although RBC transfusion might be life-saving for patients with haemorrhagic shock, uncertainty remains about the best early transfusion strategy in other patients. Our study suggests that blood transfusion could be harmful for those patients whose predicted risk of death is low. However, as our study was observational, important biases cannot be ruled out, and we cannot claim a causal link. Therefore, this hypothesis should be prospectively evaluated in a randomised controlled trial.

## Supporting Information

Table S1 **Baseline characteristics of included participants and those with missing data.**
(DOCX)

Text S1 **Development and validation of the CRASH-2 prognostic model.**
(DOCX)

## Abbreviations

OR: odds ratio
RBC: red blood cell
