# Supplementary material for: Red Blood Cell Transfusion and Mortality in Trauma Patients: Risk-Stratified Analysis of an Observational Study
Source: PLoS Med. 2014 Jun 17;11(6):e1001664. doi: 10.1371/journal.pmed.1001664 (PMC4060995; doi:10.1371/journal.pmed.1001664)
Supplement: Table S1 — Baseline characteristics of included participants and those with missing data. (DOCX) [file pmed.1001664.s001.docx]

|  |  | **Non-missing** | **Missing** |
| --- | --- | --- | --- |
| **Total** |  | **19857** | **270** |
| **Country income** | High  Middle  Low  Missing | 378 (1.9%)  19176 (96.6%)  303 (1.5%)  - | 36 (13.3%)  232 (85.9%)  2 (0.7%)  0 |
| **Tranexamic acid** | Placebo  Active  Missing | 9923 (50.0%)  9934 (50.0%)  - | 144 (53.3%)  126 (46.7%)  0 |
| **Time since injury** | ≤3 hours  >3 hours  Missing | 13273 (66.8%)  6584 (33.2%)  - | 212 (80.9%)  50 (19.1%)  8 |
| **Age (years)** | Median (IQR)  Missing | 31 (24 to 43)  - | 30 (25 to 40)  0 |
| **Systolic blood pressure (mmHg)** | Median (IQR)  Missing | 93 (80 to 110)  - | 50 (40 to 90)  28 |
| **Respiratory rate (per min)** | Median (IQR)  Missing | 22 (20 to 26)  - | 23 (20 to 25)  186 |
| **Heart rate (per min)** | Median (IQR)  Missing | 105 (90 to 120)  - | 110 (82 to 125)  137 |
| **Glasgow coma scale** | Median (IQR)  Missing | 15 (11 to 15)  - | 10 (3 to 12)  23 |
| **Penetrating injury** | No  Yes  Missing | 13461 (67.8%)  6396 (32.2%)  0 | 144 (53.3%)  126 (46.7%)  0 |

**Baseline characteristics of included participants and those with missing data.**
