# Supplementary material for: Red Blood Cell Transfusion and Mortality in Trauma Patients: Risk-Stratified Analysis of an Observational Study
Source: PLoS Med. 2014 Jun 17;11(6):e1001664. doi: 10.1371/journal.pmed.1001664 (PMC4060995; doi:10.1371/journal.pmed.1001664)

**TEXT S1:**

**Development and validation of the CRASH-2 prognostic model**

**Development**

Sample

We included patients from the Clinical Randomisation of an Antiﬁbrinolytic in Signiﬁcant Haemorrhage (CRASH-2) trial. The trial included 20,127 trauma patients with, or at risk of, significant bleeding, within 8 hours of injury, and was undertaken in 274 hospitals in 40 countries.

Outcome

The primary outcome was all cause mortality. Patient outcomes were recorded at discharge, death in hospital or 28 days after injury, whichever occurred first.

Predictors

Variables analysed as potential predictors were taken from the patient entry form completed prior to randomisation. Variables included in the CRASH-2 trial entry form can be divided into:

1. Patient demographic characteristics: age and sex.
2. Injury characteristics: type of injury and time from injury to randomization.
3. Physiological variables: Glasgow Coma Scale (GCS), systolic blood pressure, heart rate, respiratory rate, and central capillary refill time.

Variable definitions

Age was recorded as a continuous variable measured in years. Type of injury had 3 categories – penetrating, blunt, or blunt and penetrating, but was analyzed as penetrating, and “blunt and penetrating”. Time from injury was recorded as a continuous variable measured in hours. The five physiological variables were recorded according to usual clinical practice. For each of these variables the value given on the entry form was the first measurement taken at hospital admission.

Analysis

We conducted complete case analysis as the amount of missing data was very low in the CRASH-2 data. All candidate predictors were included initially in the multivariable logistic regression. Analyses were adjusted for treatment by including treatment allocation as a covariate in the models. We also included a variable for economic region (i.e. low, middle or high income country as defined by the World Bank). We used logistic regression models with random intercepts by country. Continuous variables were initially analyzed as linear terms. Departure from linearity was assessed graphically and by adding quadratic and cubic terms into the model. Interactions by age and by type of injury were specifically explored. Time since injury was dichotomized into less or more than 3 hours as the effect of this variable was reasonably well captured by using it as binary. We conducted a backward stepwise approach. We first included all potential prognostic factors and interaction terms that were considered plausible by users. These interactions included all potential predictors with type of injury, time since injury and age. We then removed, one at a time, terms for which there were no strong evidence of an association judged according to the p-values (< 0.05) from the Wald test. Each time we calculated a log-likelihood ratio test to check the term removed did not have a big impact in the model. Eventually we reached a model were all terms were statistically significant.

The formula for the final model was

Log (Odds) = -4.333 + U_j_ + 0.742*MIC + 1.224*LIC – 0.140*TXA - 0.289*AGE – 0.085*AGE^2^ + 0.015*AGE^3^ – 0.128*SBP + 0.028*SBP^2^ + 0.002*SBP^3^ - 0.231*GCS + 0.005*GCS^2^

Uj ~ N(0, SD) Random effect for each country, where estimated SD=0.75

MIC: Middle Income Countries (0=No, 1 =Yes)

LIC: Low Income Countries (0=No, 1 =Yes)

TXA: Tranexamic Acid (0=No, 1 =Yes)

HSI: Hours since injury (0=less than 3 hours, 1 =3 hours or more)

SBP: (Systolic blood pressure -120) / 10

**External validation**

For the external validation we used the data from TARN. Membership is voluntary and includes 60% of hospitals receiving trauma patients in England and Wales and some hospitals in Europe. Data are collected on patients who arrive at hospital alive and meet any of the subsequent criteria: death from injury at any point during admission, stay in hospital for longer than 3 days, require intensive or high dependency care, require inter-hospital transfer for specialist care.

Patients with isolated closed limb injuries and patients over 65 years old with isolated fractured neck of femur or pubic ramus fracture were excluded. The physiological data available in TARN are identical to that in CRASH-2 in that for every patient the heart rate, systolic blood pressure, GCS, respiratory rate and capillary refill time on arrival is entered by the hospital data co-ordinators. For each patient the volume of blood loss is estimated. This is done by allocating an estimated percentage of total volume of blood lost to each injury code in the AIS dictionary by blinded, then consensus, agreement from two emergency physicians. This estimation is based on previous work on blood loss in specific injuries.16

Adult (age > 15 yrs at the time of injury) patients presenting between 2000 and 2008 to TARN participating hospitals were selected. The definition used in the CRASH-2 trial of significant haemorrhage was not available; therefore, we only selected patients with an estimated blood loss of at least 20% who we considered would be clinically comparable with the CRASH-2 patients.

For the validation in the TARN dataset we conducted multiple imputations to substitute the missing values of the predictors included in the prognostic model using the procedure of imputation by chained equations (ICE) in Stata (StataCorp. 2009. Stata Statistical Software: Release 11. College Station, TX: StataCorp LP). We applied the coefficients of the model developed in CRASH-2 with the estimated UK intercept to the five imputed datasets of TARN, obtaining five predictions of mortality for each patient in TARN. We then averaged over these five predictions to calculate calibration and discrimination.

**Performance**

The model showed a good internal validity, with a C statistic of 0.84 and good calibration except in very high risk patients for whom the model over predicted risk (figure).

Internal validation using bootstrapping showed a minimal decrease of the C statistic from 0.836 to 0.835. This indicates that there is very low over-optimism in the model development.

For the external validation we used the same variables as included in the derivation model except hours since injury as this variable had a very large number of patients with missing data. Discrimination was good (C statistic 0.88), and calibration satisfactory (figure).

Figure: Internal and external calibration of the prognostic model by predicted risk


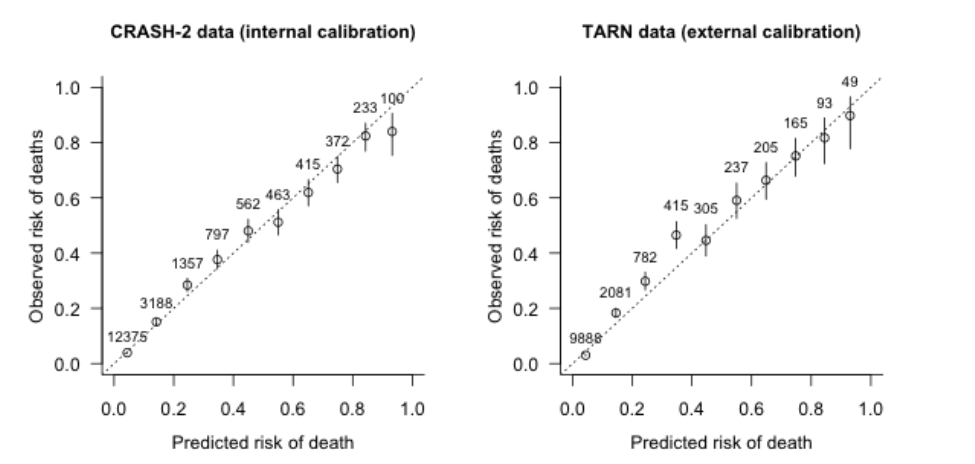

Supplement: Text S1 — Development and validation of the CRASH-2 prognostic model. (DOCX) [file pmed.1001664.s002.docx]
